# Nontuberculous Mycobacteria in Respiratory Tract Infections, Eastern Asia

**DOI:** 10.3201/eid1703100604

**Published:** 2011-03

**Authors:** Sami Simons, Jakko van Ingen, Po-Ren Hsueh, Nguyen Van Hung, P.N. Richard Dekhuijzen, Martin J. Boeree, Dick van Soolingen

**Affiliations:** Author affiliations: Radboud University Nijmegen Medical Centre, Nijmegen, the Netherlands (S. Simons, J. van Ingen, P.N.R. Dekhuijzen, M.J. Boeree);; National Institute for Public Health and the Environment (RIVM),; Bilthoven, the Netherlands (J. van Ingen, D. van Soolingen);; National Taiwan University Hospital, Taipei, Taiwan (P.-R. Hsueh);; National Hospital of Tuberculosis and Respiratory Diseases, Hanoi, Vietnam (N.V. Hung)

**Keywords:** Atypical mycobacteria, Mycobacterium, infections, Asia, respiratory tract infections, review, tuberculosis and other mycobacteria, bacteria, synopsis

## Abstract

To characterize the distribution of nontuberculous mycobacteria (NTM) species isolated from pulmonary samples from persons in Asia and their association with pulmonary infections, we reviewed the literature. *Mycobacterium avium* complex bacteria were most frequently isolated (13%–81%) and were the most common cause of pulmonary NTM disease (43%–81%). Also pathogenic were rapidly growing mycobacteria (*M. chelonae, M. fortuitum, M. abscessus*). Among all NTM isolated from pulmonary samples, 31% (582/1,744) were considered clinically relevant according to American Thoracic Society diagnostic criteria. Most patients were male (79%) and had a history of tuberculosis (37%). In Asia, high prevalence of rapidly growing mycobacteria and a history of tuberculosis are distinct characteristics of pulmonary NTM disease. This geographic variation is not well reflected in the American Thoracic Society criteria for NTM infections and could be incorporated in future guidelines.

Nontuberculous mycobacteria (NTM) are common in the environment and have been isolated worldwide (*1*). They are increasingly recognized as pathogens in humans. Pulmonary disease is the most common manifestation (*2*) and is thought to result from aerosol inhalation. Because of their omnipresence in the environment, isolation of NTM from the respiratory tract does not, per se, indicate NTM disease. Therefore, the American Thoracic Society (ATS) has established diagnostic criteria to help distinguish between contamination and true NTM disease (*1*).

Although NTM are present worldwide, much of the literature on pulmonary NTM disease comes from industrialized countries, mainly Europe, North America, and Japan. Differences in the NTM species distribution in clinical samples and disease have been noted among these regions (*3*); by extrapolation, these characteristics in other parts of the world probably differ as well.

Asia has a long history of NTM research, both clinical and fundamental. Tsukamura et al. have reported on pulmonary NTM infections in Japan dating back to the early 1970s (*4*). Yet because many studies were not reported in English, knowledge of the distribution of NTM species in Asia is limited. However, these data will enhance our understanding of NTM diversity between and within species and their association with NTM disease in humans. For this reason, we searched the literature on clinical NTM isolation and disease from different regions in Asia and compared our findings with previously published data from other regions.

## Literature Search

From March 2009 to December 2009, we searched PubMed (www.ncbi.nlm.nih.gov/pubmed) for English-language articles about nontuberculous mycobacteria in Asia. The search strategy was as follows: “mycobacteria, atypical” [MeSH] AND “Asia” [MeSH] OR “atypical mycobacterium infections” [MeSH] AND “Asia” [MeSH]. We chose the term Asia to incorporate the following countries: Brunei, Cambodia, East Timor, Indonesia, Lao People’s Democratic Republic, Malaysia, Myanmar, Philippines, Singapore, Thailand, Vietnam, Bangladesh, Bhutan, India, China, Hong Kong, Japan, South Korea, Mongolia, and Taiwan.

We found 256 citations. If we considered the abstract to be relevant, we obtained a full copy of the article; we contacted authors if full-text articles could not be retrieved. Furthermore, the reference sections were screened for other eligible citations. We considered 67 articles to be relevant, of which 37 were excluded for the following reasons: 11 were case reports, 7 referred to disseminated NTM infections, 6 were not in English (5 in Japanese, 1 in Chinese), 6 did not concern pulmonary NTM, 4 were reviews, and 3 represented neither epidemiologic data nor clinical cases. From the remaining 30 articles (*4**–**33*), the following data were abstracted for this review: country, research setting, NTM species, clinical features of the patients, and radiographic data. All articles were screened to determine whether ATS diagnostic criteria for the determination of clinical relevance of NTM isolations applied (*1*). Cases consistent with the ATS diagnostic criteria were considered clinically relevant.

## Search Results

We identified 30 English-language articles about the epidemiology and clinical relevance of NTM isolates in Asia (*4**–**33*). We found data from China (*5*), Hong Kong (*6*), India (*7**–**13*), Japan (*4**,**14**–**20*), South Korea (*21**–**24*), Singapore (*25*), Taiwan (*26**–**29*), and Thailand (*30**–**33*). Most articles used a combination of methods to identify NTM species. Biochemical and phenotypic analysis (n = 11) were used most frequently, followed by molecular tools (n = 4) or a combination of both (n = 5). From 10 articles, the exact methods of species identification could not be determined. The use of identification methods differed over time; biochemical and phenotypic analyses were mostly used during 1966–1990 (6 of 7 studies), whereas use of molecular tools increased during 1990–2009 (Table 1). The number of different species isolated did not differ between the 2 periods. However, most species identification by biochemical testing depended heavily on 1 study from India (*11*).

**Table 1:** Nontuberculous mycobacteria isolated in Asia, 1971–2007*

Species	Before 1990 (n = 1,205)†	After 1990 (n = 7,614)†
*Mycobacterium abscessus*	–	X
*M. aichiense*	X	–
*M. asiaticum*	X	–
*M. avium complex*	X	X
*M. celatum*	–	X
*M. chelonae*	X	X
*M. flavescens*	X	X
*M. fortuitum*	X	X
*M. gastri*	X	X
*M. gordonae*	X	X
*M hemophilum*	X	–
*M. kansasii*	X	X
*M. malmoense*	X	–
*M. marinum*	X	X
*M. neoarum*	X	–
*M. parafortuitum*	X	–
*M. phlei*	X	X
*M. scrofulaceum*	X	X
*M. simiae*	X	X
*M. smegmatis*	X	X
*M. szulgai*	X	X
*M. terrae*	X	X
*M. tokaiense*	X	–
*M. triviale*	X	X
*M. thermophilum*	X	–
*M. thermoresistibile*	X	–
*M. ulcerans*	X	–
*M. vaccae*	X	X
*M. xenopi*	X	X

*Data derived from references *4–33*. X, isolated; –, not isolated. †Proportions of identification methods used (biochemical:molecular) 6:1 before 1990, 5:8 after 1990.

### Epidemiology

**Figure 1:**
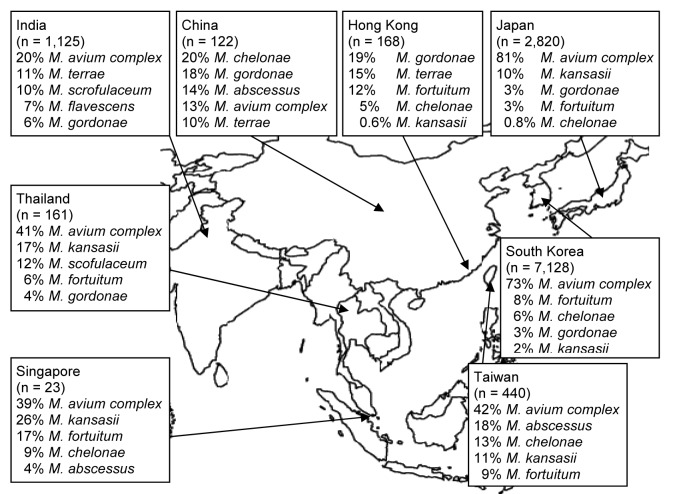
Five most prevalent nontuberculous mycobacteria species found in respiratory specimens, regardless of clinical relevance, Asia, 1971–2007. Data from (*4*–*13*,*17*–*22*,*25*–*33*).

Regardless of clinical relevance, 25 articles reported on NTM isolates from pulmonary samples (Figure 1) (*4**–**13**,**17**–**22**,**25**–**33*). In general, *Mycobacterium avium* complex (MAC) was most frequently (67%) isolated, although it predominated in northeastern Asia (South Korea and Japan). Exact species identification of MAC (now *M. avium*, *M. intracellulare*, *M. chimaera*, *M. colombiense*, *M. vulneris*, *M. marseillense*, *M. bouchedurhonense*, and *M. timonense*) (*34**,**35*) was not performed, thereby hampering a more detailed analysis.

Rapidly growing mycobacteria ([RGM] *M. fortuitum* complex, *M. abscessus, M. chelonae*) were frequently identified in pulmonary samples from Taiwan, China, and Singapore (*5**,**25**–**29*). The overall isolation of RGM in this part of Asia was 16%, making RGM the second most frequently isolated species in this region. Other species frequently encountered were *M. kansasii* (4%) and *M. gordonae* (3.5%). *M. malmoense*, a species regularly encountered in northwestern Europe, was found only 9 times in a single study of 1,000 NTM isolates from India (*11*). Also, *M. xenopi*, which is frequently encountered in Canada and England (*3*), was isolated only 10 times (of 11,987 isolates) and mainly during studies from India (*7**,**11**,**13**,**21**,**27*).

### Clinical Relevance

The clinical relevance of pulmonary NTM isolates (Table 2) was mentioned in 10 articles covering a total of 1,744 patients (*6**,**16**,**21**,**23**,**25**,**28**–**30**,**32**,**33*). No articles covered the clinical relevance of NTM in China. Different criteria were used to define clinical relevance, but all criteria comprised a combination of clinical, bacteriologic, and radiographic criteria. Only 4 studies reported a priori use of ATS criteria (*1*). According to ATS criteria, for 31% (582/1,744) of patients, pulmonary NTM isolates were considered clinically relevant. Relevance varied widely across regions and studies. For example, a study of patients in an intensive care unit in Taiwan found that only 9% of NTM isolates were clinically relevant (*28*); however, in a study of HIV-infected patients in Thailand, relevance rose to 76% (*30*).

**Table 2:** Clinical relevance of NTM species isolated from pulmonary samples collected in Asia, by country, 1971–2007*

Study area	No. patients	Patients for whom NTM infection was considered clinically relevant, %	Criteria used (year of revision)	Reference
Hong Kong	168	17	ATS criteria (1990)	(*6*)
Japan	357	76†	ATS criteria (1997)	(*16*)
South Korea	794	17	ATS criteria (1997)	(*21*)
South Korea	23	65‡	BTS criteria (1999)	(*23*)
Singapore	23	65	Compatible signs and symptoms, >3 positive sputum specimens, and radiographic features of recent lung disease	(*25*)
Taiwan	111	9	ATS criteria (1990)	(*28*)
Taiwan	169	28§	NTM in >2 samples, new radiographic lesions ,and no other pathogens (definite) or other concomitant bacteria (probable NTM) found	(29)
Thailand	33	76	NTM in sample(s); compatible signs, symptoms, and radiographic features; and no other explanation	(*30*)
Thailand	24	29	Continued NTM isolation, progressive pulmonary disease, and worsening radiographic lesions	(*32*)
Thailand	42	71	Repeated isolation of high numbers of NTM and presence of compatible disease process	(*33*)

*NTM, nontuberculous mycobacterium; ATS, American Thoracic Society (*1*); BTS, British Thoracic Society. †Only *Mycobacterium avium* complex bacteria were included in this study. ‡Only *M. kansasii* was included. §14% if only definite NTM were included.

**Figure 2:**
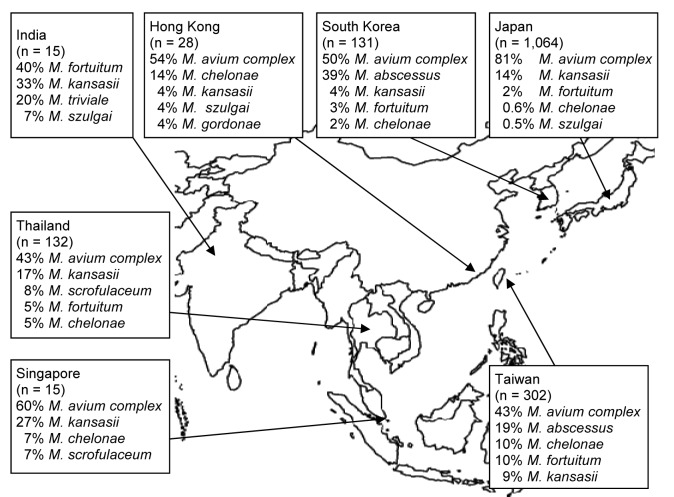
Five most common nontuberculous mycobacteria species causing pulmonary infections, Asia, 1971–2007. Data from (*4*,*6*,*10*,*13*,*17*,*19*,*21*,*25*,*27*–*33*).

Figure 2 shows the distribution of mycobacterial species among patients with definite pulmonary NTM disease in Asia, according to ATS criteria. MAC was the most frequently reported (68% of all cases) cause of NTM disease; RGM were second (14% of all cases). Prevalence of RGM pulmonary infections ranged from 2.6% (Japan) to 44% (South Korea). *M. malmoense* and *M. xenopi* were not reported as causative species in any of these studies in Asia.

**Figure 3:**
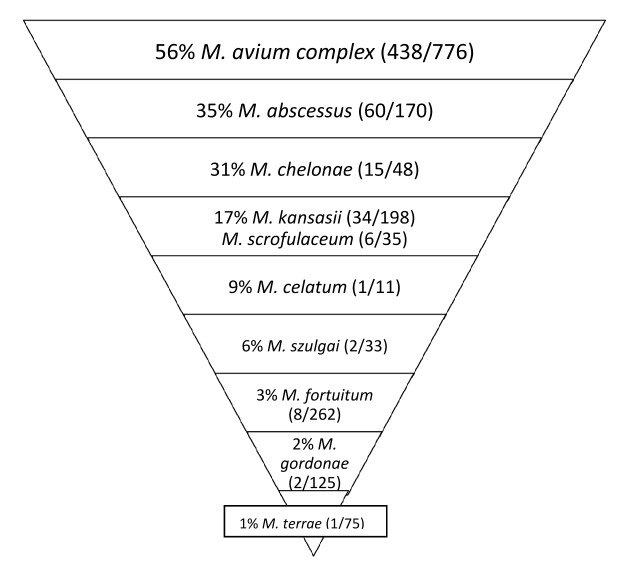
Clinical relevance of pulmonary nontuberculous mycobacterium (NTM) isolates, Asia, 1971–2007. Relevance per species was defined as percentage of patients with pulmonary NTM isolates meeting the American Thoracic Society criteria. Species reported infrequently, i.e., <5×, are not shown. Data from (*6*,*16*,*17*,*21*,*23*,*25*,*29*,*32*,*33*).

We found some discrepancies between isolation frequency (Figure 1) and clinical relevance (Figure 2). For example, in Hong Kong *M. gordonae* was found in 19% of pulmonary isolates regardless of clinical relevance but in only 4% of cases of pulmonary NTM infection. In contrast, in India, higher prevalence of *M. szulgai* was found (1% in all pulmonary samples vs. 7% of all causes of pulmonary NTM). We therefore investigated the clinical relevance of the various NTM species separately, measuring clinical relevance per species by the percentage of patients meeting ATS criteria (Figure 3). Most clinically relevant species were MAC (56%), followed by *M. abscessus* (35%) and *M. chelonae* (31%). *M. fortuitum*, *M. gordonae*, and *M. terrae* were infrequently reported (1%–3%) as clinically relevant. The varying clinical relevance per species might partially explain the differences between Figure 1 and Figure 2.

### Clinical Signs and Radiographic Features

We found information on clinical signs for 689 patients and radiographic data for 1,044 patients (*10*,*12*,*14*–*16*,*20*,*21*,*23*–*25*,*27*–*30*,*33*). Most patients with pulmonary NTM infections were male (543/689). Because of incomplete data, mean age could not be calculated; 8 studies reported mean ages of 50–70 years. Other characteristics are shown in Table 3. One third of patients had a history of tuberculosis (TB) (252/689). HIV co-infection was less prevalent among patients with localized pulmonary NTM infections (15/280). Clinical signs of NTM disease mimicked those typical of TB: most frequently chronic cough (255/268), followed by hemoptysis (82/268), fever (47/268), and weight loss (40/268). Radiographically, most patients had structural lung disease; 39% (405/1,044) had cavitations and 44% (461/1,044) had bronchiectasis.

**Table 3:** Clinical and radiographic characteristics for patients with pulmonary nontuberculous mycobacteria infections in Asia, 1971–2007*

Characteristic	No. (%) patients
Concurrent conditions, n = 689	
Malignancy, hematologic or solid	79 (11)
Gastrointestinal disease	40 (6)
HIV infection, n = 280 tested	15 (5)
Chronic corticosteroid treatment	18 (3)
Diabetes mellitus	18 (3)
Renal disease	13 (2)
Previous lung disease, n = 689	
Tuberculosis	252 (37)
Chronic obstructive pulmonary disease	62 (9)
Bronchiectasis	47 (7)
Clinical signs, n = 268	
Chronic cough	255 (95)
Hemoptysis	82 (31)
Fever	47 (18)
Weight loss	40 (15)
Radiographic findings, n = 1,044	
Cavitation	405 (39)
Nodular	559 (54)
Bronchiectasis	461 (44)

*Data from (*10*,*12*,*14*–*16*,*20*,*21*,*23*–*25*,*27*–*30*,*33*).

## Discussion

NTM isolation and disease in Asia have several features. First, a substantial percentage (31%) of patients from whom pulmonary NTM were isolated had clinically relevant NTM disease. This finding is similar to the 33% and 25% found in studies in Canada and the Netherlands, respectively (*2**,**3*). In Asia, NTM may cause substantial pulmonary disease; differences in clinical relevance exist among species (Figure 3), as previously observed (*2*).

Second, MAC was the main cause of pulmonary NTM infection (68% of cases) in Asia. In a key article in 2002, Marras and Daley reviewed the prevalence of pulmonary NTM disease in the world (*3*). They noted a predominance of MAC among the causative agents of pulmonary NTM disease in Asia. Data from Asia were, however, scarce, and their conclusion was mainly based on 1 study (*6*). Our study supports their conclusion of the predominance of MAC in Asia, which is consistent with its predominance in other parts of the world, namely, North America and most parts of Europe (*3*).

Third, we found that in some regions in Asia, RGM are a major cause of pulmonary NTM disease. This finding contrasts with studies of NTM in other parts of the world (*3*). In a surveillance study from the Netherlands for instance, RGM caused only 3% of all pulmonary NTM infections (*2*). In the United States, this percentage is ≈5% (*36*). In the present review, RGM was found to generally cause 14% of pulmonary NTM infections, but in 3 countries (India, Taiwan, South Korea) this percentage rose to >30% of infections. The fact that RGM were frequently found in pulmonary samples (Figure 1) could reflect higher environmental exposure of RGM in Asia and, hence, higher isolation frequency. The predominance of RGM species may be the result of laboratory practices as well. Ethnic factors may also contribute to susceptibility to different species; i.e., Asian persons could be more susceptible to RGM infection.

Contrary to the high frequency of isolation of RGM species, *M. malmoense* and *M. xenopi*, frequently seen in other parts of the world, were not seen as causative species in any of the studies from Asia. *M. xenopi* has been associated with hot water systems (*1*); as a result, it might be expected to be more rare in Asia, where the water delivery infrastructure is less developed than that in Europe and North America.

A fourth feature of pulmonary NTM disease in Asia—compared with Europe and North America—was the relatively high percentage of patients with a history of TB. This finding might merely reflect the higher incidence of TB in Asia, or it could reflect higher clinician awareness in Asia, such that physicians order *Mycobacterium* spp. cultures in former TB patients with coughing and hence find a relatively higher number of NTM isolates. Alternatively, it could reflect a true predilection of NTM for patients with structural lung disease (*1*) associated with a higher susceptibility to mycobacterial infection in general. The role of TB in the pathogenesis of pulmonary NTM disease is controversial; structural lung damage by a TB infection renders the host vulnerable to NTM disease (*1*), but there are also clues that exposure to TB infers cross-protection to NTM disease (*37*).

Our study has some limitations. The major limitation is the language restriction. The inclusion of languages other than English would probably have increased precision. For instance, during our literature search we came across 5 articles, published as abstracts in PubMed, on NTM infections in Japan. Although certain aspects of these data were already published in the English-language articles we included, we did not have the means to include these non–English-language studies. Nevertheless, our study illustrates the distribution of NTM infections from different geographic areas in Asia and will increase knowledge of the distribution and relevance of NTM species in Asia.

Another limitation is the long time span of the included studies. Because they ranged from 1969 to 2008, they used different decontamination, culture, and identification methods. Data should therefore be considered with caution because of the variety of laboratory procedures used by the several authors to isolate and identify NTM. First, different sample decontamination protocols may determine the yield of NTM by selecting for certain NTM species and inhibiting others (*38*). Second, the introduction of more sensitive liquid media probably increased NTM isolation and perhaps selected for specific species (*26*). Finally, and foremost, the major differences in identification methods used in the studies introduce important biases. Because the taxonomy of NTM has been changing in recent years (*37**,**39**,**40*), the different NTM identification methods used in the various studies might have influenced our results; use of molecular tools to identify the historical isolates would probably result in different, more detailed, identifications, especially among the MAC, the *M. simiae* group, and the RGM (*37**,**39**,**40*). We did note increased use of molecular tools for NTM identification over time (Table 1), which will, over the next few years, provide us with a more up-to-date overview of NTM species distribution in Asia.

In conclusion, despite the limitations of language and species identification methods, we have described the scale of human pulmonary NTM infections in eastern Asia. MAC bacteria were not only the most prevalent NTM found in pulmonary samples, but they were also the most common cause of pulmonary NTM disease in this geographic region. Distinct epidemiologic and clinical characteristics of pulmonary NTM disease in Asia were found: many patients had a history of TB, and RGM were a frequent cause of pulmonary NTM infections. These distinct characteristics of pulmonary NTM disease in this part of the world are not well reflected in the current ATS criteria on NTM infections and could be incorporated in future guidelines.
